# Highly Sensitive Capacitive Pressure Sensor Based on MWCNTs/TiO_2_/PDMS with a Microhemispherical Array and APTES-Modified Interface

**DOI:** 10.3390/polym18010012

**Published:** 2025-12-20

**Authors:** Yijin Ouyang, Jianyong Lei, Shuge Li, Guotian He, Songxiying He

**Affiliations:** 1 School of Mechanical Engineering, Chongqing University of Technology, Chongqing 400054, China; jack_sparrow00@163.com; 2 Chongqing Institute of Green and Intelligent Technology, Chinese Academy of Sciences, Chongqing 400714, China; leijianyongn@126.com (J.L.); lishuge@cqcet.edu.cn (S.L.); heguotian@cigit.ac.cn (G.H.); 3 Chongqing Institute of the University of Chinese Academy of Sciences, Chongqing University, Chongqing 400044, China; 4 School of Smart Health, Chongqing Polytechnic University of Electronic Technology, Chongqing 401331, China; 5 Chongqing Luban Robotics Technology Research Institute Co., Ltd., Chongqing 400799, China

**Keywords:** composites, flexible sensors, surface modification, carbon nanotubes, microstructure

## Abstract

The rapid advancement of humanoid robotics has spurred researchers’ interest in flexible sensors for wide linear range detection. In response, we report a capacitive flexible pressure sensor based on a multi-walled carbon nanotubes/titanium dioxide/polydimethylsiloxane (MWCNTs/TiO_2_/PDMS) composite. A micro-hemispherical structure array formed on the composite surface via a templating method reduces the initial capacitance value. Modified carbon nanotubes (F-MWCNTs) were prepared using 2 wt%, 5 wt% and 10 wt% γ-aminopropyltriethoxysilane (APTES), significantly enhancing dispersion and interfacial bonding strength. The synergistic effect of microstructures and MWCNTs surface functionalization further enhances sensing performance. The F-MWCNTs/TiO_2_/PDMS pressure sensor modified with 2 wt% APTES exhibits outstanding sensing capabilities: it demonstrates dual-stage sensitivity across a broad linear range of 0–95 kPa (0–13 kPa segment: 1.89 ± 0.49 kPa^−1^; 13–95 kPa segment: 7.08 ± 0.63 kPa^−1^), with a response time of 200 milliseconds, maintaining stability over 2500 cyclic loadings. In practical application exploration, this sensor has demonstrated strong adaptability, confirming its significant potential in micro-pressure detection, wearable electronics, and array sensing applications.

## 1. Introduction

Recently, the rapid development of humanoid robotics has drawn more attention from researchers to flexible pressure sensors. The core function of a pressure sensor, as a detection device, is to convert an applied physical pressure signal into a measurable electrical signal output. According to the different sensing principles, the main types include piezoresistive, capacitive, piezoelectric, triboelectric and inductive. Among them, flexible capacitive pressure sensors are characterized by low power consumption, fast response, small drift, and high linearity and therefore have a promising future in automotives [1,2], robotics [3,4], healthcare [5,6], aerospace [7,8] and wearable electronic devices [9,10].

Carbon nanotubes (CNTs) are nanoscale tubular materials formed by carbon atoms through sp^2^ hybridization. Due to their exceptional strength and outstanding electrical conductivity, they have been widely adopted in the field of flexible pressure sensors [11,12]. However, in the face of complex application scenarios, the properties of CNTs by themselves are usually unable to meet the variable conditions of use. Therefore, it has been a research hotspot to enhance the performance of sensors by modifying CNTs. Zhang [13] and colleagues used silane coupling agent (SCA) to modify CNTs, and the diffusion of CNTs into PDMS through the solvation/permeation method proved to improve the sensitivity of the composites. He et al. [14] employed the simultaneous use of silane coupling agent (KH550) and sodium dodecylbenzene sulfonate (SDBS) modification to improve the dispersion and compatibility of multi-walled CNTs in polyurethane (PU). Although the incorporation of modified CNTs drastically reduces the hysteresis error and repeatability error of the sensors, a large nonlinear error still exists. The group of Hyeyoung [15] reported a dodecyl amine (DDA)-based modification of MWCNTs/PDMS dielectric elastomers, which enhances the reliable dielectric properties of the composites. However, alkylamines are toxic and will cause harm to the human body.

Sensitivity is an important indicator of sensing performance, and it has been shown that structural design, such as pyramidal, hemispherical, petal, fabric structure, and porous microstructures, can enhance sensitivity [16,17,18,19,20]. In 2010, the team of Bao [21] first integrated microstructure PDMS as a dielectric layer into a flexible pressure sensor and achieved up to 0.55 kPa-1high sensitivity with subtle pressure detection capability. Ji et al. [22] fabricated gradient micro dome structure (GDA) templates using a micro forming technique combined with conductive CNTs to achieve ultra-wide extension of the linear range and improved sensitivity. Shao et al. [23] used thermally expandable microspheres added to a silica matrix to form a porous structure that improves the compressibility of the elastomer, thus enhancing the sensor sensitivity. Thermally expandable microspheres consist of a hydrocarbon core and a thermoplastic shell, which expand during heating due to hydrocarbon-induced expansion of the thermoplastic shell. However, the density of the expanded hydrocarbons decreases, upwelling occurs, and the porous structure is concentrated in the surface layer or the upper layer. Yang et al. [24] used the template method to introduce holes into the pyramidal dielectric structure. The results showed that the sensitivity of pyramid sensors with the presence of porous structures was approximately twice as high as that of pure solid pyramid sensors. However, it is worth noting that nonlinearity is observed in high-voltage sensing due to stress concentration at the tip of the pyramid.

This paper proposes a preparation strategy for dielectric composites integrating chemical modification and microstructure design, applied to capacitive flexible pressure sensors. During the chemical modification stage, MWCNTs were treated with an APTES-ethanol mixed solution under weakly acidic conditions, followed by efficient cleaning and separation using an ultrafiltration membrane filtration system. After drying and grinding, the F-MWCNTs powder is obtained for subsequent use. For microstructure construction, microforming technology is employed to etch a polytetrafluoroethylene (PTFE) template. By injecting the F-MWCNTs/TiO_2_/PDMS composite slurry, followed by curing and demolding processes, an elastic force-sensitive element featuring a micro-hemispherical array structure is successfully fabricated. This modification process effectively enhances the interfacial bonding strength between MWCNTs and the PDMS matrix while improving MWCNT dispersion within the matrix, significantly boosting the composite material’s mechanical properties and electrical characteristics. Experimental results demonstrate that the 2 wt% APTES-modified F-MWCNTs/TiO_2_/PDMS flexible pressure sensor exhibits outstanding sensing performance, including high sensitivity, a wide linear range, and excellent cyclic stability. This sensor shows great application potential in practical scenarios such as micro-pressure detection and array sensing.

## 2. Experiments

### 2.1. Materials

Polydimethylsiloxane (Sylgard 184, comprising silicone rubber and curing agent in a 10:1 ratio) was supplied by Dow Chemical Company (Midland, MI, USA). Rutile-type titanium dioxide with an average diameter of 5 nm was supplied by Xindun Alloy Welding Spray Co., Ltd. (Tangshan, China). TEM characterisation of the pristine form of TiO_2_, as shown in Figure S1. Hydroxylated multi-walled carbon nanotubes (Purity: 95%, Outer diameter: 8–15 nm, Length: 3–12 μm, Wall count: 5–20, Hydroxyl content: approximately 2 wt%) supplied by Suzhou Carbon Fund Graphene Technology Co., Ltd. (Suzhou, China).

Reagents for modifying MWCNTs: γ-aminopropyltriethoxysilane (APTES) and deionised water were supplied by Kangjin New Materials Technology Co., Ltd. (Dongguan, China) and Elder Technology Co., Ltd. (Hangzhou, China). The ethanol solution with a concentration of 99.7% was provided by Sinopharm Chemical Reagent Co., Ltd. (Beijing, China). The acetic acid solution with a concentration of 99.5% was provided by Zhiyuan Chemical Reagent Co., Ltd. (Tianjin, China).

### 2.2. Modified MWCNTs

**Figure 1:**
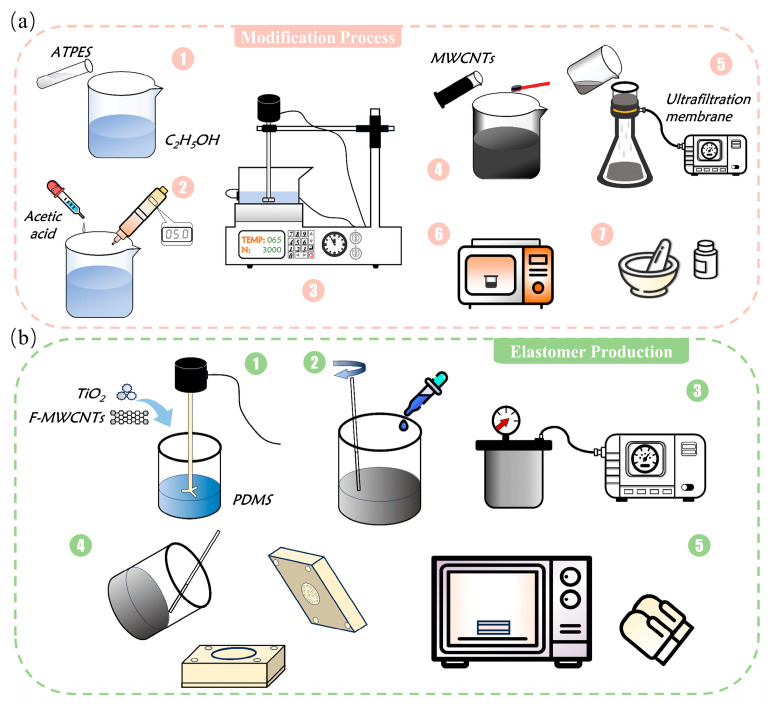
(**a**) Process Flow Chart for Surface Modification of Carbon Nanotubes. (**b**) Flowchart for the Preparation of Composite Material Force-Sensitive Elements.

As shown in Figure 1a, weigh 2 g, 5 g, and 10 g of APTES and add them to an ethanol solution to prepare APTES ethanol solutions with mass fractions of 2 wt%, 5 wt%, and 10 wt%, respectively. To accelerate the hydrolysis of -OC_2_H_5_ in APTES to form reactive Si-OH groups, ~15 g of glacial acetic acid was added dropwise to the mixed solution, adjusting the pH to a weakly acidic range of 4.5–5.0. The mixture was then subjected to slow stirring at 65 °C for 30 min. The reaction process is as follows:

(1) $${NH}_{2}{\left({CH}_{2}\right)}_{3}Si{\left({OCH}_{2}{CH}_{3}\right)}_{3}+{3H}_{2}O\overset{H^{+}}{\Rightarrow }{{{NH}_{2}\left({CH}_{2}\right)}_{3}Si\left(OH\right)}_{3}+3{CH}_{3}{CH}_{2}OH$$

Ethanol reduces the concentration of free water in the system, preventing the instantaneous complete hydrolysis of APTES into large amounts of highly reactive silanol groups. This inhibits the tendency of high silanol concentrations to condense and form gel-like polymers. Simultaneously, as a solvent possessing both hydrophilic and lipophilic properties, it facilitates the dilution of pure water to further dissolve APTES. Acetic acid acts as a catalyst in this reaction. The acidic conditions promote alkoxy protonation, enhancing the electrophilicity of the silicon atom and facilitating nucleophilic attack by water molecules. Subsequently, the three alkoxy groups undergo stepwise hydrolysis, ultimately yielding trihydroxysilane (NH_2_(CH_2_)_3_Si(OH)_3_).

**Figure 2:**
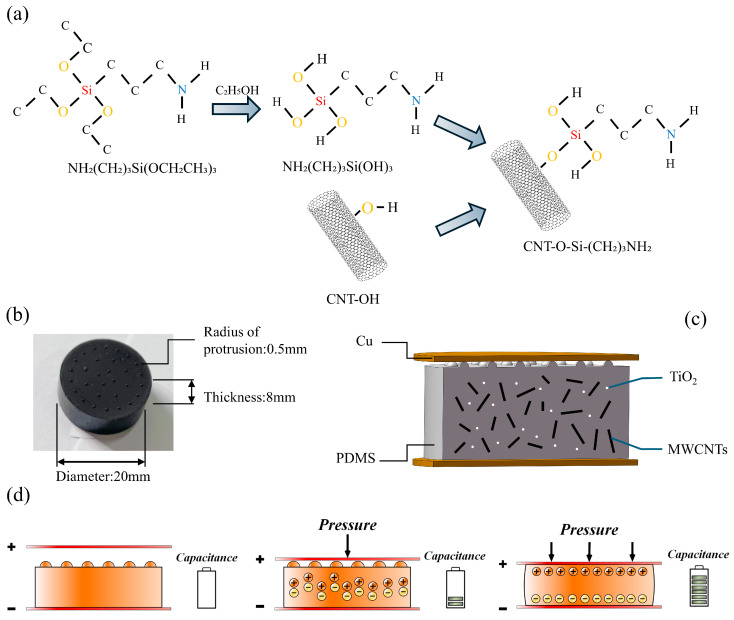
(**a**) Schematic diagram of the modification principle. (**b**) Schematic diagram of the sensor principle. (**c**) Component configuration diagram of the sensor. (**d**) Photograph of composite material.

At 25 °C, 5 g of hydroxylated MWCNTs were immersed in the activated APTES solution prepared in the preceding step for 1 h. During this process, Si-OH groups generated by APTES hydrolysis reacted with -OH groups on the carbon nanotube surface, forming stable Si-O-C covalent bonds. This achieved chemical anchoring of APTES molecules onto the CNT surface (Figure 2a). The reaction process is as follows:

(2) $$CNTOH+{NH}_{2}{\left({CH}_{2}\right)}_{3}Si{\left(OH\right)}_{3}\Rightarrow CNTOSi{\left(CH_{2}\right)}_{3}{NH}_{2}+H_{2}O$$

Subsequently, the mixture was filtered using a polyethersulfone ultrafiltration membrane with a pore size of 0.02 μm to thoroughly remove residual acidic solution and unreacted APTES. The carbon nanotubes were rinsed with copious amounts of deionised water until the wash solution became neutral. The washed modified carbon nanotubes were placed in an oven and dried at 80 °C for 24 h. Finally, grinding yielded a uniform material, resulting in the preparation of APTES-modified carbon nanotubes, designated as F-MWCNTs.

### 2.3. Preparation of Force Sensitive Elements

10 g of PDMS, 0.2 g of TiO_2_, and 1.3 g of F-MWCNTs were weighed into a mixer. (The original batch was measured volumetrically, with mass calculated retrospectively based on the measured density). The components were mixed via mechanical stirring at 500 rpm for 3–5 min until a uniform and stable composite slurry was formed (Figure 1b). Subsequently, the curing agent was injected at a mass ratio of 10:1 relative to the PDMS matrix and mixed thoroughly by hand. Following vacuum degassing, the mixture was injected into a custom-made mold. Figure S2 illustrates the fabrication process for fully filling small hemispherical cavities. The mould is fabricated from engraved PTFE, featuring a top plate surface with uniformly distributed hemispherical dimples of 0.5 mm radius arranged in a circular pattern. The number and distribution of dimples are detailed in Figure S3. The sealed mould was placed in an oven for curing, employing a stepwise heating programme to prevent stress concentration-induced cracking: 10 min at 40 °C for preheating, 20 min at 60 °C for heating, and 40 min at 80 °C for curing. After demoulding, the force-sensitive element of the sensor was obtained (Figure 2b). The control group of unmodified MWCNTs incorporated 0.96 g of MWCNTs by mass, with all other raw material quantities, process parameters, and curing procedures identical to those described above.

### 2.4. Characterization

The chemical properties of the CNTs samples were characterized using a Fourier transform microinfrared imaging spectrometer (Nicolet IS20, Thermo Fisher Scientific, Inc., Waltham, MA, USA); the distribution of CNTs and TiO_2_ in the PDMS was observed by scanning electron microscopy (S-4800, Hitachi, Inc., Tokyo, Japan); the mechanical properties of the composite elastomers were tested simultaneously using a microcomputer-controlled electronic universal testing machine (FBS200N, Furbs Detection Equipment Co., Ltd., Xiamen, China) and an LCR digital bridge (UC2836B, Youce Electronic Technology Co., Ltd., Changzhou, China). The universal testing machine operates in load control mode with a test frequency of 10 kHz, a load range of 0 to 30 N, and a compression rate of 0.02 mm/min. Sensitivity was characterized by the following equation:

(3) $$S=\frac{\frac{∆C}{C_{0}}}{∆P}$$

where $\frac{∆C}{C_{0}}$ indicates the relative capacitance change of the sensor under pressure, ∆P represents pressure change. ∆C is the change in capacitance with pressure and $C_{0}$ is the initial capacitance value.

To assess the effectiveness of linear fitting, the coefficient of determination *R*^2^ is introduced to measure the goodness of fit of the regression model, defined as:

(4) $$R^{2}=1-\frac{{SS}_{res}}{{SS}_{tot}}=1-\frac{\sum_{i=1}^{n}{\left(o_{i}-o_{i}^{*}\right)}^{2}}{\sum_{i=1}^{n}{\left(o_{i}-o_{a}\right)}^{2}}$$

$R^{2}$ ranges from 0 to 1, with higher values indicating better model fit. $o_{i}$ denotes the actual output value of the i-th data point, $o_{i}^{*}$ denotes the theoretical output value for the i-th data point, while $o_{a}$ represents the average of all actual output values.

## 3. Results and Discussion

### 3.1. Sensing Principles

**Figure 6:**
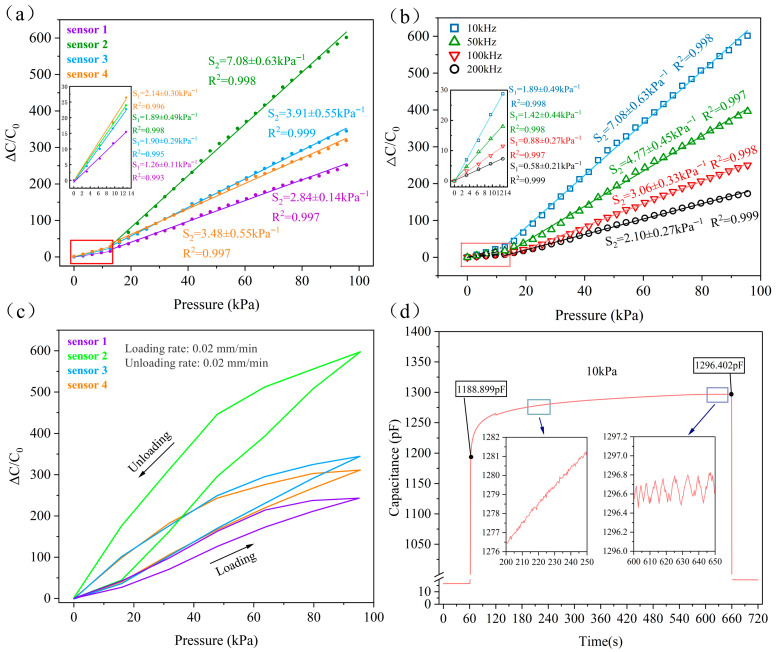
(**a**) Sensitivity of Sensors 1, 2, 3, and 4 at a test frequency of 10 kHz. (**b**) Sensitivity of Sensor 2 at test frequencies of 10 kHz, 50 kHz, 100 kHz, and 200 kHz. (**c**) Single loading-unloading curves for Sensors 1, 2, 3, and 4, demonstrating sensor hysteresis. (**d**) Capacitance signal output of Sensor 2 under 10 kPa pressure sustained for 10 min.

As shown in Figure 2c, the sensor adopts a sandwich configuration. The upper and lower electrode layers consist of pure copper sheets, while the dielectric layer utilizes PDMS as a flexible matrix. By incorporating MWCNTs and rutile-type TiO_2_ with a high intrinsic dielectric constant as reinforcing phases, an efficient synergistic dielectric network is jointly constructed. The combination of PDMS/TiO_2_/MWCNTs constructs a multi-layered interfacial polarisation structure. The synergistic effect of these numerous heterogeneous interfaces (PDMS/TiO_2_, TiO_2_/MWCNTs, PDMS/MWCNTs) substantially enhances the effective dielectric constant. As shown in Figure S4, when TiO_2_ or MWCNTs were incorporated into PDMS alone, neither sample exhibited a significant enhancement in sensitivity during the polarisation reaction. However, Figure 6 demonstrates that the PDMS/TiO_2_/MWCNTs composite exhibits markedly improved sensitivity, attributable to the synergistic enhancement effect at the heterogeneous interface.

Capacitive sensors are essentially a class of capacitors that are charged and discharged by moving the electrode plates, and the positive and negative electrons in the medium are brought close to the electrode plates of opposite properties under the action of the external potential difference of the electrons of their respective properties, producing an electric dipole moment to store the electricity, which is known as the polarization reaction [25]. Microstructure design is one approach to enhancing sensor sensitivity [16]. Its presence allows air to enter the narrow gap between the electrode plate and the composite material. Air possesses a relatively low dielectric constant, leading to a decrease in the initial capacitance value and an increase in the capacitance change value, thereby enhancing the sensor’s sensitivity. As shown in Figure 2d, when external pressure is applied, the hemispherical microstructure first contacts the electrode plate. The originally electrically neutral atoms within the force-sensitive unit generate a small electric dipole moment, resulting in a small capacitance value. As pressure continues to be applied, the microstructure flattens. The entire surface of the force-sensitive unit comes into close contact with the electrode plate, eliminating air. The polarization reaction intensifies, generating a larger electric dipole moment and resulting in a higher capacitance value. Therefore, under the influence of the microstructure, the sensor exhibits an output characteristic with two distinct slopes.

### 3.2. Modification Effect

**Figure 3:**
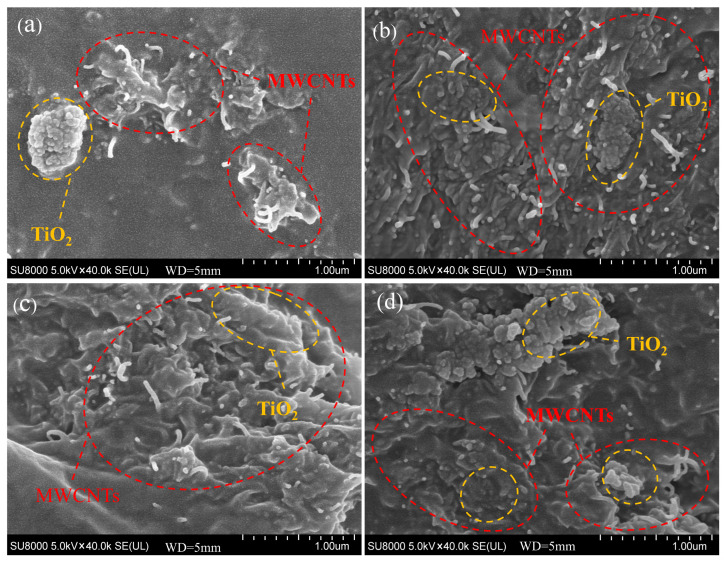
SEM cross-sectional images: (**a**–**d**) represent the MWCNTs/TiO_2_/PDMS composite, and the F-MWCNTs/TiO_2_/PDMS composites modified with 2 wt%, 5 wt% and 10 wt% APTES, respectively.

**Figure 4:**
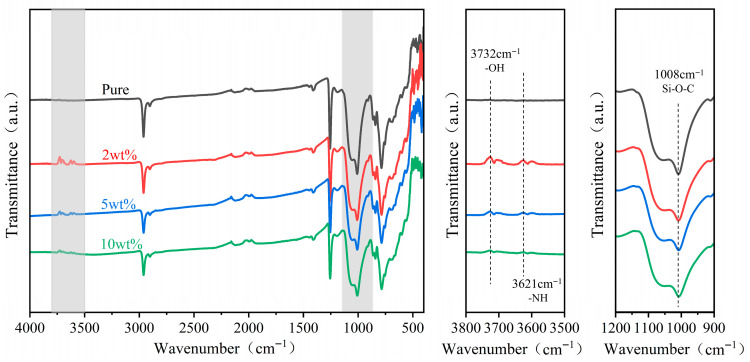
FTIR spectra of MWCNTs, 2 wt%, 5 wt%, and 10 wt% APTES-modified F-MWCNTs.

**Figure 5:**
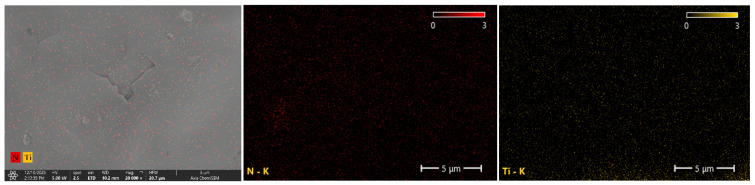
EDS image: Sample of F-MWCNTs/TiO_2_/PDMS modified with 2 wt% APTES.

Figure 3 shows the scanning electron micrograph of the force-sensitive composite material, illustrating the distribution of unmodified MWCNTs, F-MWCNTs, and TiO_2_ within the PDMS substrate at the microscopic level. Compared to the state before APTES introduction (Figure 3a), the dispersion of MWCNTs within the PDMS significantly improved after APTES incorporation (Figure 3b,c,d), with a marked reduction in agglomeration. MWCNTs uniformly surround the periphery of TiO_2_, increasing interfacial connections between different phases through physical and chemical interactions, which will enhance their synergistic performance. To verify whether APTES had genuinely grafted onto the MWCNTs, we analysed the samples using Fourier Transform Infrared Spectroscopy (FTIR). As shown in Figure 4, compared to the unmodified sample, the modified sample exhibited new characteristic absorption peaks at 3732 cm^−1^, 3621 cm^−1^, and 1008 cm^−1^. The peak at 3732 cm^−1^ is attributed to the isolated (Si-OH) group formed upon APTES hydrolysis, whilst the peak at 3621 cm^−1^ likely originates from strong interactions between the terminal (-NH_2_) group of APTES and the (OH) groups on the TiO_2_ surface. Furthermore, the stretching vibration peak at 1008 cm^−1^ corresponds to the Si-O-C bond, confirming successful grafting of APTES onto the MWCNT surface. The effective chemical bridging interface formed by APTES between carbon nanotubes and titanium dioxide is anticipated to positively influence the electrical and sensing properties of the dielectric polymer composite material. Figure 5 displays the Energy-Dispersive Spectrometer (EDS) analysis of the F-MWCNTs/TiO_2_/PDMS sample modified with 2 wt% APTES. Following modification, nitrogen and titanium elements appear uniformly distributed across the sample surface.

### 3.3. Performance

According to the experimental procedure, four sensor groups were fabricated using MWCNTs and F-MWCNTs with APTES mass fractions of 2 wt%, 5 wt%, and 10 wt%, designated as Sensors 1–3, and 4, respectively. The quantitative data for each group represents the average of five independent sensors, with error bars controlling for ±one standard deviation. Figure 6a summarizes the two-stage sensing characteristics exhibited by each sensor at a test frequency of 10 kHz. Among these, Sensor 2 exhibits high sensitivity (7.08 ± 0.63 kPa^−1^) and high linearity within the 13~95 kPa pressure range. As shown in Figures S5 and S6, the micro-hemispherical structure not only alters the single-segment sensing characteristics but also reduces the initial capacitance. APTES not only mitigates CNT agglomeration and enhances the interfacial bonding between CNTs, TiO_2_, and the PDMS matrix, but also improves the composite’s electrical and mechanical properties, thereby ultimately amplifying its capacitive effect and sensitivity. However, excessive coupling agent leads to multi-layer adsorption on the CNT surface, hindering direct bonding with the substrate and consequently reducing interfacial strength. This explains why Sensor 3 and Sensor 4, despite containing more APTES, exhibit lower sensitivity than Sensor 2.

Figure 6b illustrates Sensor 2’s sensing performance across different test frequencies within the same pressure range. At 100 kHz, the sensor sensitivity is only 3.06 ± 0.33 kPa^−1^ (13–95 kPa), while at 200 kHz, sensitivity further decreases to 2.10 ± 0.27 kPa^−1^. Figure S7 shows that the unmodified, flat-bottomed force sensor without microstructures exhibits the same trend as the former, with sensitivity decreasing as the test frequency increases. This again demonstrates that capacitive sensor measurements at high frequencies will exhibit distortion.

Hysteresis performance was characterised by the following equation:

(5) $$H=\frac{A_{U}-A_{L}}{A_{L}}\times 100\%$$

$A_{U}$ and $A_{L}$ are the area of the curves under loading and unloading, respectively.

We hypothesise that this relates to dielectric relaxation. At high frequencies, the electric field changes extremely rapidly, preventing the interface polarisation from fully responding and thereby reducing its contribution to sensitivity. As shown in Figure 6c, the hysteresis of Sensors 1–4 were 8.216%, 11.537%, 10.748%, and 10.054%, respectively. APTES enhances the interfacial adhesion between MWCNTs and PDMS, increasing the viscoelasticity of the composite material. During rebound cycles, this tight interface adhesion impedes the composite’s return to its original shape.

Creep refers to the process where strain in solid materials increases over time under constant stress. We conducted a static pressure test on Sensor 2 at 10 kPa for 10 min. The results in Figure 6d show that the capacitance value rose sharply after pressure application, subsequently stabilising gradually. The capacitance change over 10 min amounted to 107.503 pF. Sensors will continue to undergo gradual deformation under sustained stress, exhibiting significant creep behaviour. This results in a slow drift of the output signal over time, severely disrupting constant force measurements. Concurrently, as the signal output fails to rapidly return to its initial value following unloading, interference is introduced into subsequent measurements. However, signal deviations caused by creep can be statically compensated using higher-order linear viscoelastic gray-box frameworks [26]. In contrast, dynamic signal drift can be mitigated through a hybrid approach combining fast dynamic time warping and multi-source domain adversarial neural networks [27].

**Figure 7:**
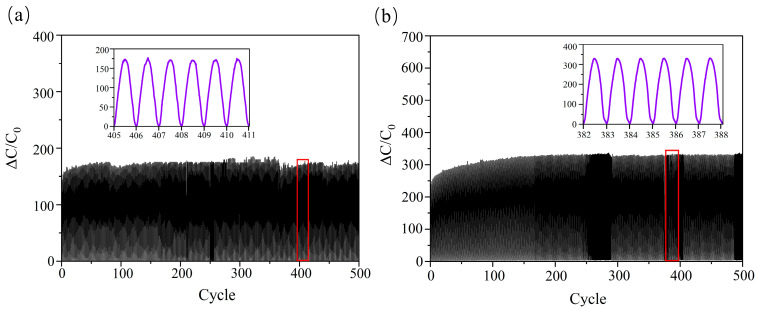
(**a**) and (**b**) show the 500-cycle loading tests for Sensor 1 and Sensor 2, respectively.

To investigate the sensors’ stability and repeatability, we subjected Sensor 1 and Sensor 2 to 500 cyclic load cycles. As shown in Figure 7a, Sensor 1 rapidly formed a peak plateau during cycling but subsequently exhibited noticeable peak jitter. The microstructure of the force-sensitive element is prone to internal dielectric damage under repeated pressure. The modified, robust interfacial bonding enhances the structure’s strength. Sensor 2 did not exhibit abrupt peak changes, demonstrating consistent electrical signal response and excellent cyclic stability (Figure 7b). Based on the results of 10,000 cycles shown in Figure S8, Sensor 2 can achieve stable cyclic loading for 500 cycles. Within the first 2000 cycles, the capacitance change rate (ΔC/C_0_) of Sensor 2 exhibits a relatively gradual trend. However, after exceeding 2500 cycles, ΔC/C_0_ begins to rise sharply and becomes unstable. Table 1 summarises the performance characteristics of all sensors discussed in this paper. Among them, Sensor 2 exhibits the most favourable overall performance. The capacitive pressure sensors fabricated in this work demonstrate competitive overall performance compared to recent studies of similar types (Table 2). Although dielectric relaxation exacerbates hysteresis, interfacial polarisation significantly enhances sensitivity, which may represent a trade-off approach for improving sensor sensitivity. By employing microstructural design and chemical modification with coupling agents, this study significantly enhances sensor sensitivity and operational linear range, offering distinct advantages in precision sensing applications.

### 3.4. Applications

**Figure 8:**
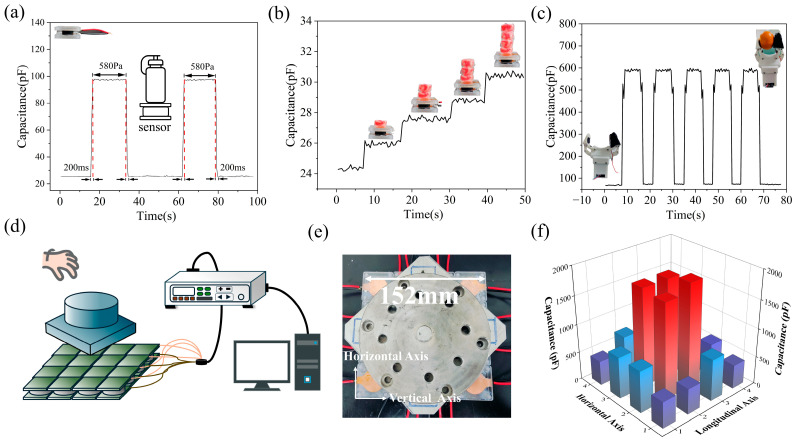
Application Testing of Sensor 2: (**a**) Response time during loading/unloading at 580 Pa pressure. (**b**) Electrical signal response under increasing micropressure conditions. (**c**) Application performance when the sensor is worn on a mechanical gripper. (**d**), (**e**), (**f**) respectively show a schematic diagram of an array sensor detection device, a physical detection image, and a 3D electrical signal response diagram.

Given its outstanding sensing performance, we selected Sensor 2 for the relevant application tests. As shown in Figure 8a, the capacitive flexible pressure sensor exhibited a fast response time of 200 ms under approximately 580 Pa of pressure loading and unloading. Figure 8b shows an experiment where Alpine candies (each approximately 65 Pa) were progressively stacked onto the sensor surface to test its electrical signal response to minute pressures. Results indicate that Sensor 2 not only exhibits a distinct electrical signal response to minute pressures but also demonstrates exceptional stability. To further evaluate the sensor’s stability in practical applications and its potential for use in wearable electronics, we mounted the sensor on a mechanical gripper to simulate the gripping motion of a human hand joint. As shown in Figure 8c, when gripping the orange at a constant angular velocity, the output signal exhibits jitter during both the mechanical claw’s closing and opening phases. This occurs because the gripping action is not instantaneous, and the orange’s soft texture also cushions the gripping force. Notably, when fully clamped, the sensor maintains a stable electrical signal without any signal drift.

Sensor arrays have broad application prospects in the fields of smart devices, electronic skin, and object recognition and reconstruction, which not only test the sensing performance of individual sensors, but also put higher requirements on the structural rationality and reliability of the whole array [34,35,36]. As shown in Figure 8d,e, we formed a 4 × 4 square array using 16 Sensor 2 units to test whether stacking metal objects could reveal the tested object’s contour by superimposing array signals. To minimize electrode plate tilting and reduce capacitance distortion, limiting springs were installed at each corner of the individual sensors. An LCR digital bridge paired with a multiplexer switch system (UC0836A, Youce Electronic Technology Co., Ltd., Changzhou, China) enabled simultaneous measurement of multiple capacitance data sets. The application results are shown in Figure 8f. The well-defined capacitance signals reconstruct the shape of the workpiece, demonstrating the array’s capability to reproduce object contours. Therefore, we conclude that the F-MWCNTs/TiO_2_/PDMS flexible capacitive force sensor modified with 2 wt% APTES exhibits promising performance and potential for practical applications.

### 3.5. Limitations and Future Work

All reported sensitivities were obtained under uncontrolled laboratory environmental conditions without temperature or humidity regulation. PDMS-based capacitive sensors are known to be humidity-sensitive, and the quantitative performance metrics reported herein may vary with environmental conditions. Therefore, validation based on actual operating temperatures and humidity levels is required prior to deployment in wearable or medical applications. Furthermore, FTIR evidence of APTES grafting (such as the Si-O-C peak at 1008 cm^−1^) merely suggests the potential presence of covalent bonding; it does not constitute definitive proof. Further validation may require methods such as thermogravimetric analysis or X-ray photoelectron spectroscopy.

## 4. Conclusions

Overall, we have developed a novel capacitive flexible pressure sensor by combining composite elastomer microstructure design with chemical modification using coupling agents. Surface modification of MWCNTs with APTES significantly improved the interfacial interaction between MWCNTs and PDMS, enhanced CNT dispersion, and further amplified the capacitive pressure effect. The capacitive force sensor utilising a composite material of F-MWCNTs/TiO_2_/PDMS modified with 2 wt% APTES exhibits reliable sensing performance, including high sensitivity of 1.89 ± 0.49 kPa^−1^ (0–13 kPa) and 7.08 ± 0.63 kPa^−1^ (13–95 kPa), a broad linear range from 0 to 95 kPa, a rapid response time of 200 milliseconds, and cyclic stability over 2500 cycles. However, high sensitivity comes at the cost of increased hysteresis and baseline drift. Moreover, due to the effects of stress, slip and diffusion occur within the material’s crystal lattice [37], and further research is required to mitigate the hysteresis phenomenon.

## Figures and Tables

**Table 1:** Performance Comparison Table for All Sensors Mentioned in the Text.

	Initial Capacitance (pF)	Max Capacitance (pF)	Sensitivity (kPa^−1^)	R^2^ Values	Hysteresis (%)
Sensor 1	14.12	3455.72	0–13 kPa: 1.26 ± 0.11 13–95 kPa: 2.84 ± 0.14	0.993 0.997	8.216%
Sensor 2	11.52	6894.48	0–13 kPa: 1.89 ± 0.49 13–95 kPa: 7.08 ± 0.63	0.998 0.998	11.537%
Sensor 3	12.02	4309.31	0–13 kPa: 1.90 ± 0.29 13–95 kPa: 3.91 ± 0.55	0.995 0.999	10.748%
Sensor 4	12.91	4083.57	0–13 kPa: 2.14 ± 0.30 13–95 kPa: 3.48 ± 0.55	0.996 0.997	10.054%

**Table 2:** Performance Comparison of Capacitive Flexible Pressure Sensors.

Materials	Sensitivity (kPa^−1^)	Linear Range (kPa)	Response Time (ms)	Stability (times)	Hysteresis (%)	Year
CNTs/PDMS	0.065	0–1700	<100	7000	-	2021 [22]
PDMS	0.56	0–20	115	1000	2.8	2021 [28]
MWCNTs/ Ecoflex	2.13	<4	<100	100	-	2022 [29]
MWCNTs /PDMS	2.012	<3	<20	1000	-	2023 [30]
PDMS	2.64	0–20	45	1200	2.5	2023 [31]
PDMS	0.132	0–630	183	5250	6.6	2024 [32]
MWCNTs/ Ecoflex	0.75	0–60	129	500	6.6	2024 [33]
F-MWCNTs/ TiO_2_/PDMS	7.08	13–95	200	500	11.537	This work

## Data Availability

The raw data supporting the conclusions of this article will be made available by the authors on request.

## Supplementary Materials

The following supporting information can be downloaded at https://www.mdpi.com/article/10.3390/polym18010012/s1, Figure S1: TEM: TiO_2_ scale characterisation; Figure S2: Schematic diagram of the microstructure design for the top template; Figure S3: Composite material filling process for moulds; Figure S4: Single-factor comparison: PDMS/TiO_2_ and PDMS/MWCNTs; Figure S5: Sensitivity comparison under different preparation processes; Figure S6: describes the microstructure changes in the initial capacitance value of composite materials; Figure S7: Sensitivity of flat bottomed composite materials at different frequencies; Figure S8: 10,000 cycles of loading for Sensor 2.

## References

[1] Andrews J.B., Cardenas J.A., Lim C.J., Noyce S.G., Mullett J., Franklin A.D. (2018). Fully Printed and Flexible Carbon Nanotube Transistors for Pressure Sensing in Automobile Tires. IEEE Sens. J..

[2] Duc C.K., Hoang V.P., Nguyen D.T., Dao T.T. (2019). A Low-Cost, Flexible Pressure Capacitor Sensor Using Polyurethane for Wireless Vehicle Detection. Polymers.

[3] Tang Z.J., Wang Z., Lu J.Q., Ma G.Q. (2019). Design of robot finger based on flexible tactile sensor. Int. J. Adv. Robot. Syst..

[4] Pei Z., Zhang Q., Yang K., Yuan Z.Y., Zhang W.D., Sang S.B. (2021). A Fully 3D-Printed Wearable Piezoresistive Strain and Tactile Sensing Array for Robot Hand. Adv. Mater. Technol..

[5] Chen S., Zhuo B., Guo X. (2016). Large Area One-Step Facile Processing of Microstructured Elastomeric Dielectric Film for High Sensitivity and Durable Sensing over Wide Pressure Range. ACS Appl. Mater. Interfaces.

[6] Yu A.X., Zhu M.Y., Chen C.K., Li Y., Cui H.X., Liu S.J., Zhao Q. (2024). Implantable Flexible Sensors for Health Monitoring. Adv. Healthc. Mater..

[7] Lü X.Z., Jiang J.A., Wang H., Gao Q.B., Zhao S.B., Li N., Yang J.Y., Wang S.L., Bao W.M., Chen R.J. (2019). Sensitivity-Compensated Micro-Pressure Flexible Sensor for Aerospace Vehicle. Sensors.

[8] Callegari S., Zagnoni M., Golfarelli A., Tartagni M., Talamelli A., Proli P., Rossetti A. (2006). Experiments on aircraft flight parameter detection by on-skin sensors. Sens. Actuators A Phys..

[9] Yang J.C., Mun J., Kwon S.Y., Park S., Bao Z.N., Park S. (2019). Electronic Skin: Recent Progress and Future Prospects for Skin-Attachable Devices for Health Monitoring, Robotics, and Prosthetics. Adv. Mater..

[10] Amoli V., Kim J.S., Jee E., Chung Y.S., Kim S.Y., Koo J., Choi H., Kim Y., Kim D.H. (2019). A bioinspired hydrogen bond-triggered ultrasensitive ionic mechanoreceptor skin. Nat. Commun..

[11] Lipomi D.J., Vosgueritchian M., Tee B.C.K., Hellstrom S.L., Lee J.A., Fox C.H., Bao Z. (2011). Skin-like pressure and strain sensors based on transparent elastic films of carbon nanotubes. Nat. Nanotechnol..

[12] Ma C., Zhou R.Y., Xie L.J. (2022). Recent advances in flexible pressure/strain sensors using carbon nanotubes. Int. J. Agric. Biol. Eng..

[13] Zhang R., Ying C., Gao H., Liu Q., Fu X., Hu S. (2019). Highly flexible strain sensors based on polydimethylsiloxane/carbon nanotubes (CNTs) prepared by a swelling/permeating method and enhanced sensitivity by CNTs surface modification. Compos. Sci. Technol..

[14] He Y., Ming Y., Li W., Li Y.F., Wu M.Q., Song J.Z., Li X.J., Liu H. (2018). Highly Stable and Flexible Pressure Sensors with Modified Multi-Walled Carbon Nanotube/Polymer Composites for Human Monitoring. Sensors.

[15] Jang H., Yoon H., Ko Y., Choi J., Lee S.-S., Jeon I., Kim J.-H., Kim H. (2016). Enhanced performance in capacitive force sensors using carbon nanotube/polydimethylsiloxane nanocomposites with high dielectric properties. Nanoscale.

[16] Li R., Zhou Q., Bi Y., Cao S., Xia X., Yang A., Li S., Xiao X. (2021). Research progress of flexible capacitive pressure sensor for sensitivity enhancement approaches. Sens. Actuators A Phys..

[17] Zeng Z.R., Li Y.F., Zhao Y.L., Yuan J., Yi L.J., Li P.L., Cheng G.J., Liu F. (2023). High sensitivity and wide range flexible piezoresistive sensor based on petal-shaped MOF-derived NiCo-NPC. Nanotechnology.

[18] Xu J., Li H.Y., Yin Y.M., Li X., Cao J.W., Feng H.F., Bao W.D., Tan H., Xiao F.Y., Zhu G. (2022). High sensitivity and broad linearity range pressure sensor based on hierarchical in-situ filling porous structure. npj Flex. Electron..

[19] Chen R., Luo T., Wang J.C., Wang R.P., Zhang C., Xie Y., Qin L.F., Yao H.M., Zhou W. (2023). Nonlinearity synergy: An elegant strategy for realizing high-sensitivity and wide-linear-range pressure sensing. Nat. Commun..

[20] Tai G.J., Wei D.P., Su M., Li P., Xie L., Yang J. (2022). Force-Sensitive Interface Engineering in Flexible Pressure Sensors: A Review. Sensors.

[21] Mannsfeld S.C.B., Tee C.K., Stoltenberg R.M., Chen H.H., Barman S., Muir B.V.O., Sokolov A.N., Reese C., Bao Z. (2010). Highly sensitive flexible pressure sensors with microstructured rubber dielectric layers. Nat. Mater..

[22] Ji B.Z.Q., Lei M., Ding S., Song Q., Gao Y.B., Li S.B., Xu Y., Zhou Y.N., Zhou B.P. (2021). Gradient Architecture-Enabled Capacitive Tactile Sensor with High Sensitivity and Ultrabroad Linearity Range. Small.

[23] Shao T., Wu J., Zhang Y., Cheng Y., Zuo Z., Lv H., Ying M., Wong C.P., Li Z. (2020). Highly Sensitive Conformal Pressure Sensing Coatings Based on Thermally Expandable Microspheres. Adv. Mater. Technol..

[24] Yang J.C., Kim J.O., Oh J., Kwon S.Y., Sim J.Y., Kim D.W., Choi H.B., Park S. (2019). Microstructured Porous Pyramid-Based Ultrahigh Sensitive Pressure Sensor Insensitive to Strain and Temperature. ACS Appl. Mater. Interfaces.

[25] Liu W.T., Sun X.Z., Yan X.Y., Gao Y.H., Zhang X., Wang K., Ma Y.W. (2024). Review of Energy Storage Capacitor Technology. Batteries.

[26] Hu Z.K., Wang Y.J., Feng K.M., Chu Z.Y., Cui J., Sun F.C. (2023). A Viscoelastic Compensator for Force Sensors with Soft Materials. IEEE Trans. Instrum. Meas..

[27] Tian C., Shao W., Li Y., Shi J., Lv F., Gao R., Wei X., Zheng W. (2026). Compensation strategy of dynamic creep drift for flexible piezoresistive sensors with historical signals. Measurement.

[28] Masihi S., Panahi M., Maddipatla D., Hanson A.J., Bose A.K., Hajian S., Palaniappan V., Narakathu B.B., Bazuin B.J., Atashbar M.Z. (2021). Highly Sensitive Porous PDMS-Based Capacitive Pressure Sensors Fabricated on Fabric Platform for Wearable Applications. ACS Sens..

[29] Liu F.H., Dai S.P., Cao J., Zhang Z.Y., Cheng G.G., Ding J.N. (2022). CNTs based capacitive stretchable pressure sensor with stable performance. Sens. Actuators A Phys..

[30] Zhang X., Dang D., Su S., Wang Z., Tong Z. (2023). A Highly Sensitive Flexible Capacitive Pressure Sensor with Wide Detection Range Based on Bionic Gradient Microstructures. IEEE Sens. J..

[31] Li J.M., Zhang J., Qin L., Lv L.Y., Liu T.X., Zhang Y.T., Dhakal R., Li X., Liu T., Li Y.Y. (2023). A flexible and highly sensitive capacitive pressure sensor with fast response based on a hierarchically micro-structured PDMS dielectric layer. J. Micromech. Microeng..

[32] Farman M., Prajesh R., Panwar D.K., Kaur M., Thouti E. (2024). Cleanroom-free fabrication of flexible capacitive pressure sensors using paintable silver electrodes on stationery paper and random microstructured polydimethylsiloxane dielectric layer. Flex. Print. Electron..

[33] Jiang C.K., Sheng B. (2024). Linear Capacitive Pressure Sensor with Gradient Architecture through Laser Ablation on MWCNT/Ecoflex Film. Polymers.

[34] Baptista F.R., Belhout S.A., Giordani S., Quinn S.J. (2015). Recent developments in carbon nanomaterial sensors. Chem. Soc. Rev..

[35] Li M., Pei Y.S., Cao Y., Chen S.J., Guo X.J. (2021). Flexible strain sensors: From devices to array integration. Flex. Print. Electron..

[36] Duan Y.H., He S.X., Wu J., Su B.L., Wang Y.S. (2022). Recent Progress in Flexible Pressure Sensor Arrays. Nanomaterials.

[37] Liu F.X., Jing X., Yang J., Mi H.Y., Feng F.Y., Liu Y.J. (2025). Recent progress in low hysteresis gels: Strategies, applications, and challenges. Nano Today.

