# Supplementary material for: Highly Sensitive Capacitive Pressure Sensor Based on MWCNTs/TiO2/PDMS with a Microhemispherical Array and APTES-Modified Interface
Source: Polymers (Basel). 2025 Dec 20;18(1):12. doi: 10.3390/polym18010012 (PMC12787593; doi:10.3390/polym18010012)
Supplement: Supplementary file 1 [file polymers-18-00012-s001.zip › polymers-4000858-supplementary.pdf]

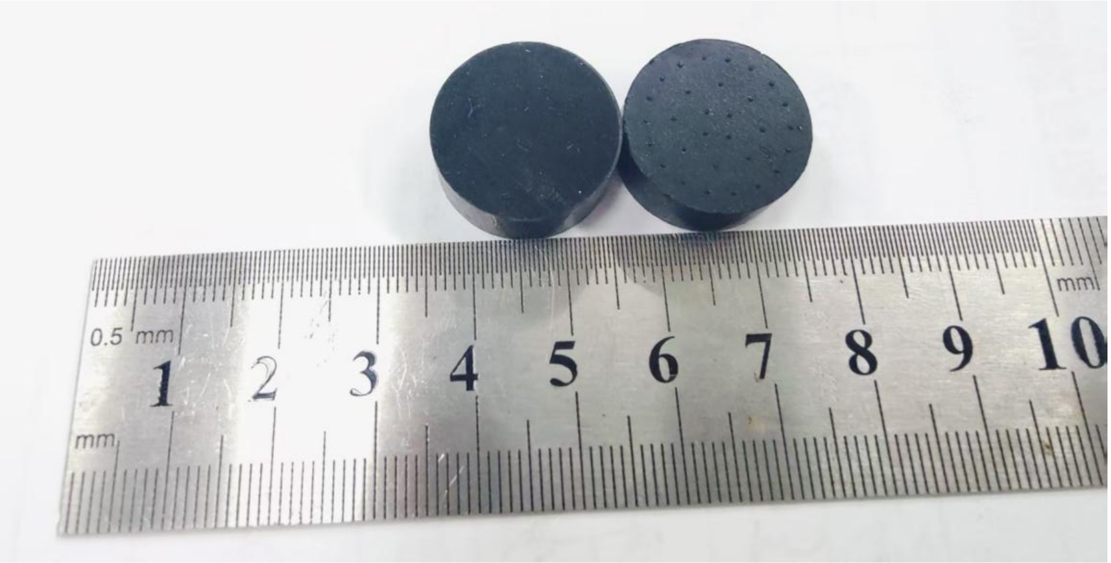


This drawing is not quoted in the text, but only to show the real size and specific structure. (flat bottom and microstructure).

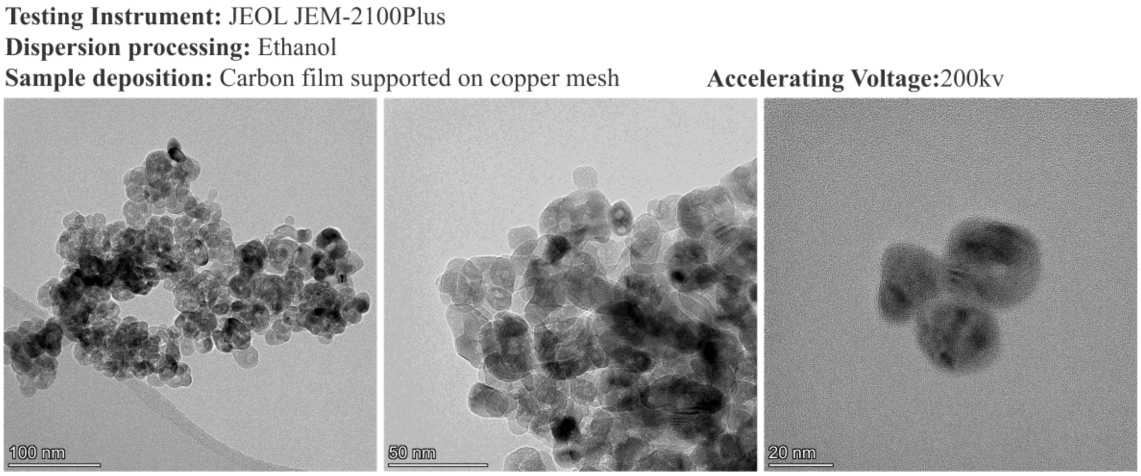


**Figure S1.** TEM: $TiO_2$ scale characterisation.

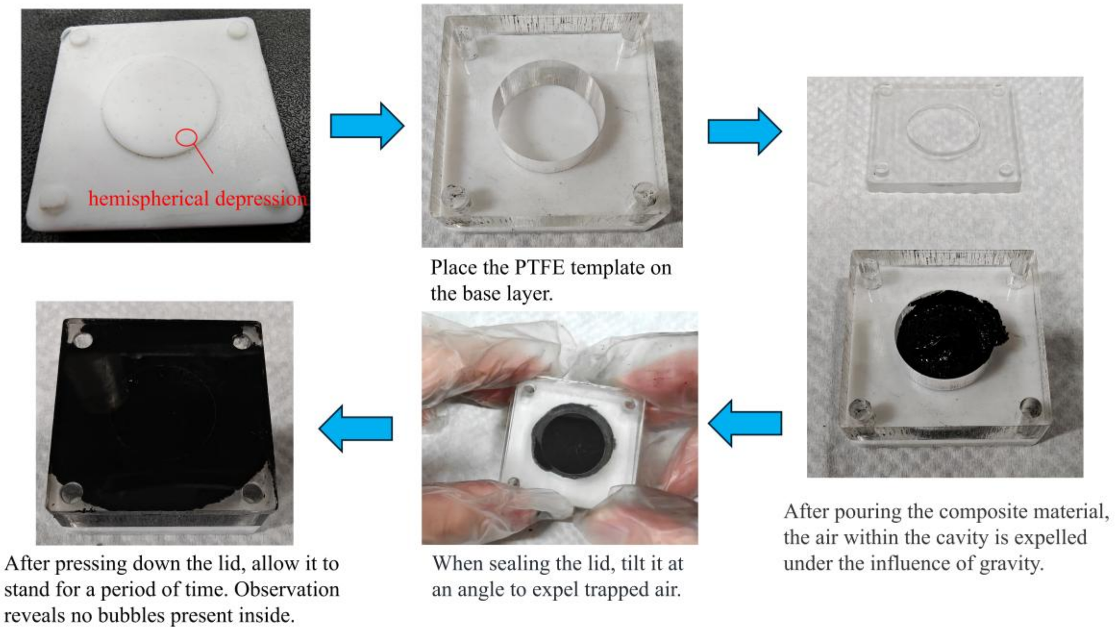


**Figure S2.** Schematic diagram of the microstructure design for the top template.

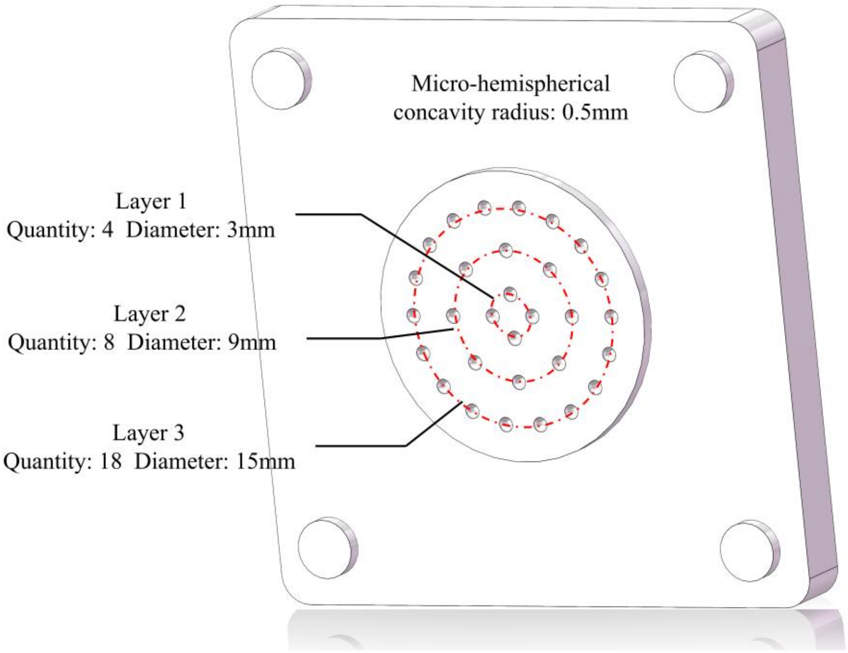


**Figure S3.** Composite material filling process for moulds.

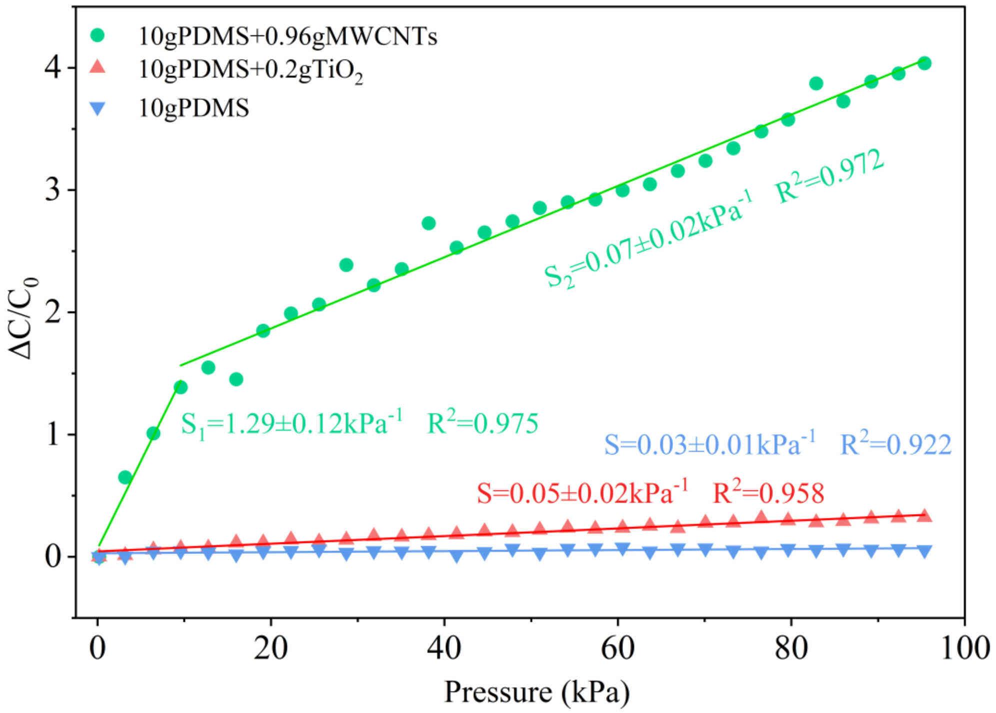


**Figure S4.** Single-factor comparison: PDMS/$TiO_2$ and PDMS/MWCNTs.

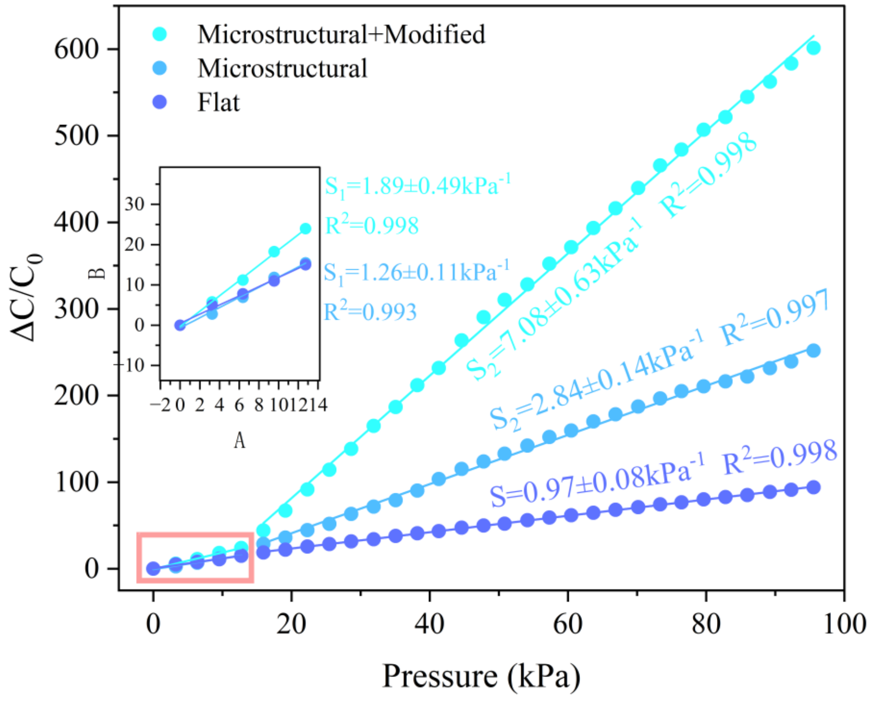


**Figure S5.** Sensitivity comparison under different preparation processes.

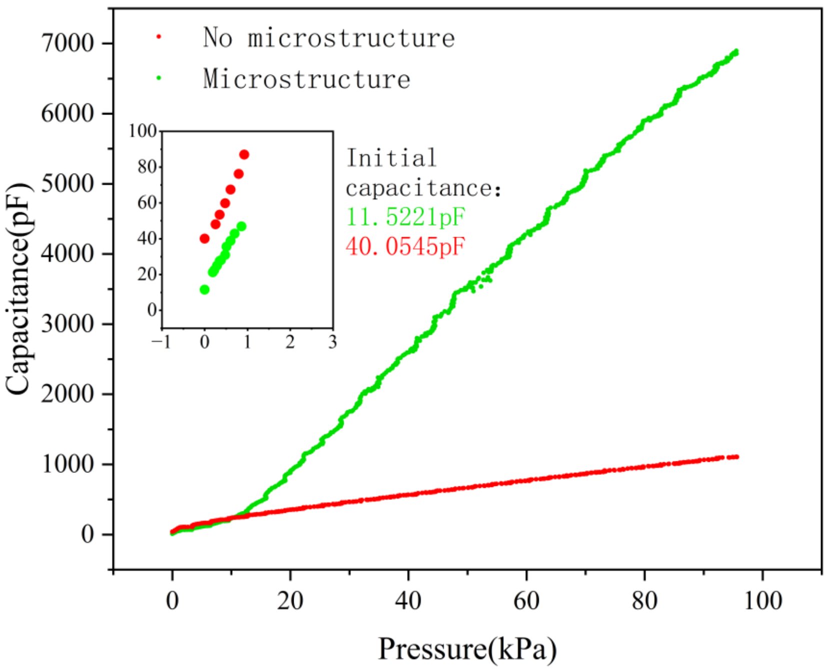


**Figure S6.** describes the microstructure changes in the initial capacitance value of composite materials.

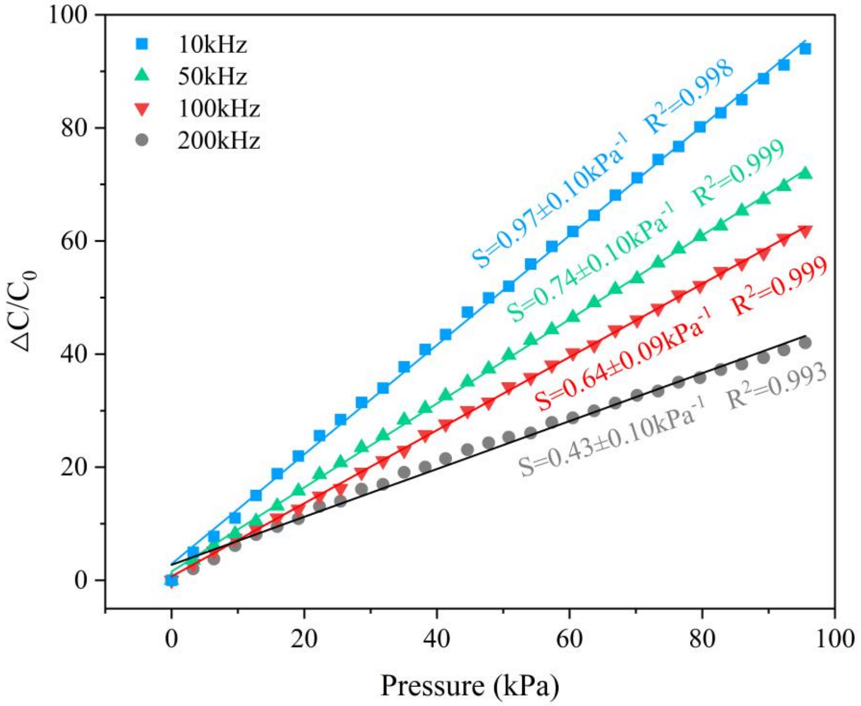


**Figure S7.** Sensitivity of flat bottomed composite materials at different frequencies

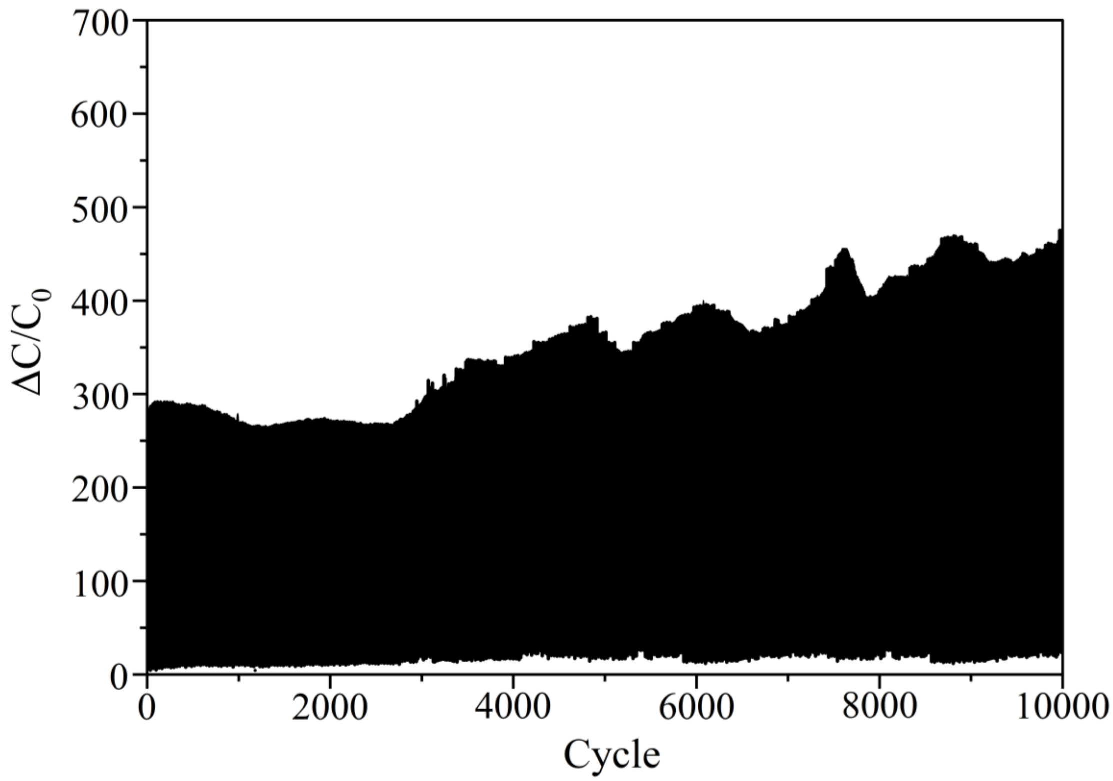


**Figure S8.** 10000 cycles of loading for Sensor 2
